# Supplementary material for: Penicillium citrinum Provides Transkingdom Growth Benefits in Choy Sum (Brassica rapa var. parachinensis)
Source: J Fungi (Basel). 2023 Mar 29;9(4):420. doi: 10.3390/jof9040420 (PMC10143594; doi:10.3390/jof9040420)
Supplement: Supplementary file 1 [file jof-09-00420-s001.zip › jof-2278166-supplementary resubmitted.pdf]

Supplementary Figure S1

Morphometric, and taxonomic identification of beneficial fungal isolates.

(a)

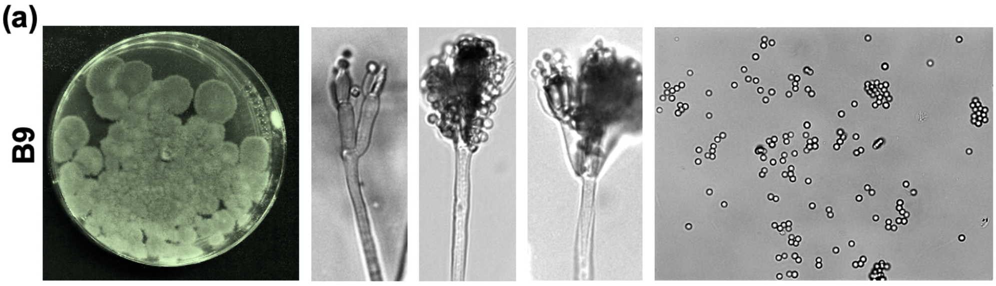

(b)

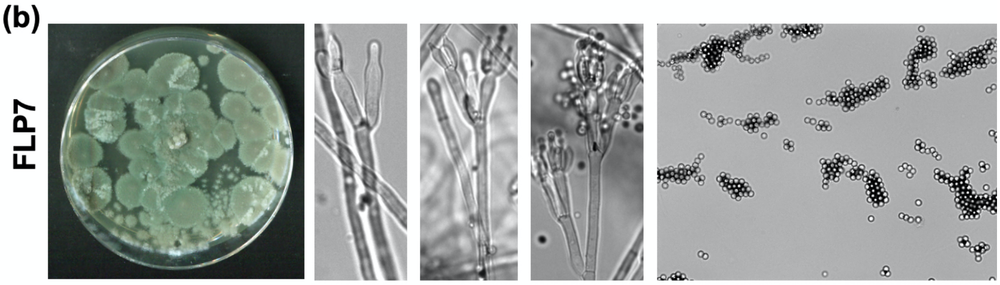

(c)

TCGGGGCCAACCTCCCACCCGTGTTGCCCGAACCTATGTTGCCTCGGCGGGCCCCGCGCCCGCCGACGGCCCCCCTGAACGCTGTCTGAAGTTG
CAGTCTGAGACCTATAACGAAATTAGTTAAAACTTTCAACAACGGATCTCTTGGTTCCGGCATCGATGAAGAACGCAGCGAAATGCGATAACTAATG
TGAATTGCAGAATTCAGTGAATCATCGAGTCTTTGAACGCACATTGCGCCCTCTGGTATTCCGGAGGGCATGCCTGTCCGAGCGTCATTGCTGCCC
TCAAGCCCGGCTTGTGTGTTGGGCCCCGTCCCCCCCGCCGGGGGGACGGGCCCGAAAGGCAGCGGCGGCACCGCGTCCGGTCCTCGAGCGTAT
GGGGCTTCGTCACCCGCTCTAGTAGGCCCGGCCGGCGCCAGCCGACCCCCAACCTTTAATTATCTCAGGTTGACCTCGGATCAGGTAGGGATACC
CGCTGAACTTAAGCATATCA

***Penicillium citrinum* isolate HT_DC16_53 internal transcribed spacer 1, 5.8S ribosomal RNA gene and internal transcribed spacer 2, complete sequence; and large subunit ribosomal RNA gene, partial sequence**
Sequence ID: MN634531.1 Length: 516 Number of Matches: 1

Alignment statistics for match #1

Score	Expect	Identities	Gaps	Strand
915 bits(495)	0.0	495/495(100%)	0/495(0%)	Plus/Plus

```
Query  1    TCGGGGCCAACCTCCCACCCGTGTTGCCCGAACCTATGTTGCCTCGGCGGGCCCCGCGCC  60
            ||||||||||||||||||||||||||||||||||||||||||||||||||||||||||||
Sbjct  8    TCGGGGCCAACCTCCCACCCGTGTTGCCCGAACCTATGTTGCCTCGGCGGGCCCCGCGCC  67

Query  61   CGCCGACGGCCCCCCTGAACGCTGTCTGAAGTTGCAGTCTGAGACCTATAACGAAATTAG  120
            ||||||||||||||||||||||||||||||||||||||||||||||||||||||||||||
Sbjct  68   CGCCGACGGCCCCCCTGAACGCTGTCTGAAGTTGCAGTCTGAGACCTATAACGAAATTAG  127

Query  121  TTAAAACTTTCAACAACGGATCTCTTGGTTCCGGCATCGATGAAGAACGCAGCGAAATGC  180
            ||||||||||||||||||||||||||||||||||||||||||||||||||||||||||||
Sbjct  128  TTAAAACTTTCAACAACGGATCTCTTGGTTCCGGCATCGATGAAGAACGCAGCGAAATGC  187

Query  181  GATAACTAATGTGAATTGCAGAATTCAGTGAATCATCGAGTCTTTGAACGCACATTGCGC  240
            ||||||||||||||||||||||||||||||||||||||||||||||||||||||||||||
Sbjct  188  GATAACTAATGTGAATTGCAGAATTCAGTGAATCATCGAGTCTTTGAACGCACATTGCGC  247

Query  241  CCTCTGGTATTCCGGAGGGCATGCCTGTCCGAGCGTCATTGCTGCCCTCAAGCCCGGCTT  300
            ||||||||||||||||||||||||||||||||||||||||||||||||||||||||||||
Sbjct  248  CCTCTGGTATTCCGGAGGGCATGCCTGTCCGAGCGTCATTGCTGCCCTCAAGCCCGGCTT  307

Query  301  GTGTGTTGGGCCCCGTcccccccGCCGGGGGGACGGGCCCGAAAGGCAGCGGCGGCACCG  360
            ||||||||||||||||||||||||||||||||||||||||||||||||||||||||||||
Sbjct  308  GTGTGTTGGGCCCCGTCCCCCCCGCCGGGGGGACGGGCCCGAAAGGCAGCGGCGGCACCG  367

Query  361  CGTCCGGTCCTCGAGCGTATGGGGCTTCGTCACCCGCTCTAGTAGGCCCGGCCGGCGCCA  420
            ||||||||||||||||||||||||||||||||||||||||||||||||||||||||||||
Sbjct  368  CGTCCGGTCCTCGAGCGTATGGGGCTTCGTCACCCGCTCTAGTAGGCCCGGCCGGCGCCA  427

Query  421  GCCGACCCCCAACCTTTAATTATCTCAGGTTGACCTCGGATCAGGTAGGGATACCCGCTG  480
            ||||||||||||||||||||||||||||||||||||||||||||||||||||||||||||
Sbjct  428  GCCGACCCCCAACCTTTAATTATCTCAGGTTGACCTCGGATCAGGTAGGGATACCCGCTG  487

Query  481  AACTTAAGCATATCA  495
            |||||||||||||||
Sbjct  488  AACTTAAGCATATCA  502
```

Figure S1: Morphometric, and taxonomic identification of beneficial fungal isolates (a) Colony morphology, and conidial characteristics for *P. citrinum* isolates B9, and FLP7 (**b**). (**c**) ITS sequence analysis and NCBI BLAST search-based confirmation of the identity of isolate B9 (and FLP7; not shown) as a strain belonging to *Penicillium citrinum.*

Supplementary Figure S2:

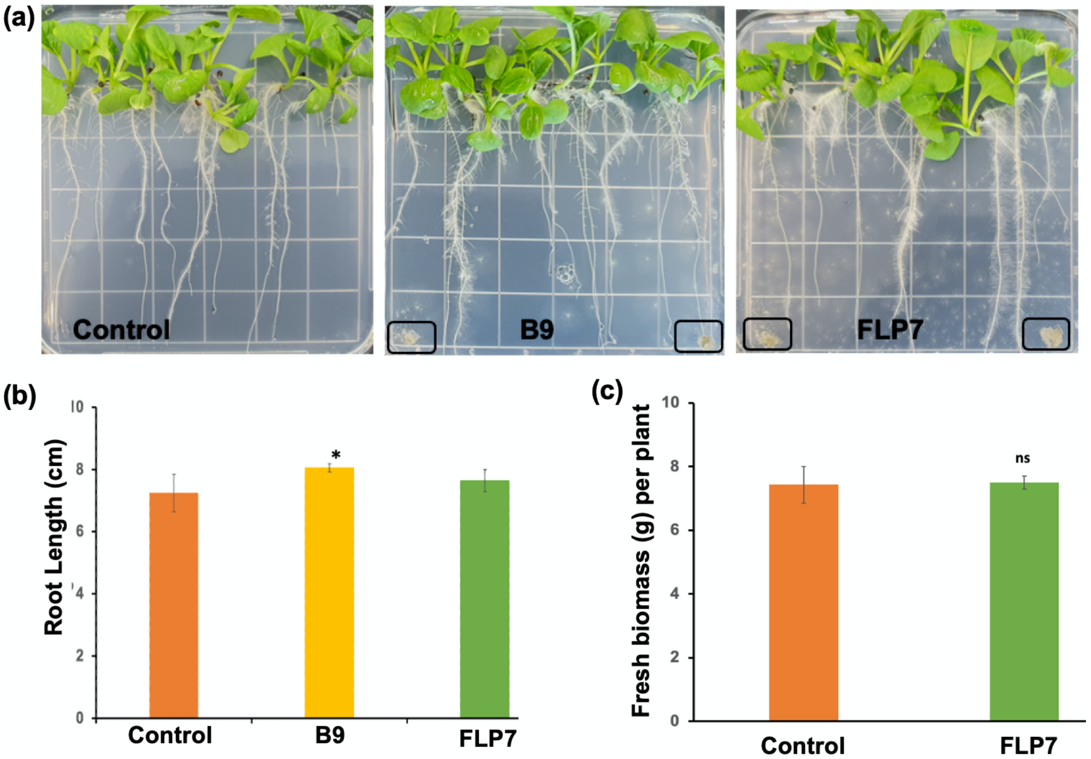

Figure S2: Effect of *P. citrinum* strain FLP7 (and/or B9) on the growth of Choy Sum (**a**) image showing growth of Choy Sum on MS medium inoculated without (control) or with the fungal plug from B9 or FLP7 strain (**b**) Average root length of seedlings at 10 days (**c**) the fresh biomass of plants grown in sterilized soil (21 days) and inoculated with FLP7 while transplanting the plants to the soil.

Supplementary Figure S3

Total Ion Chromatograms (TIC) for the chemical standards for 2 important classes of phytohormones i.e Gibberellins and Cytokinins.

(a) Total ion chromatogram for Gibberellic acid standards (GA_1, GA_3, GA_{20}, GA_4)

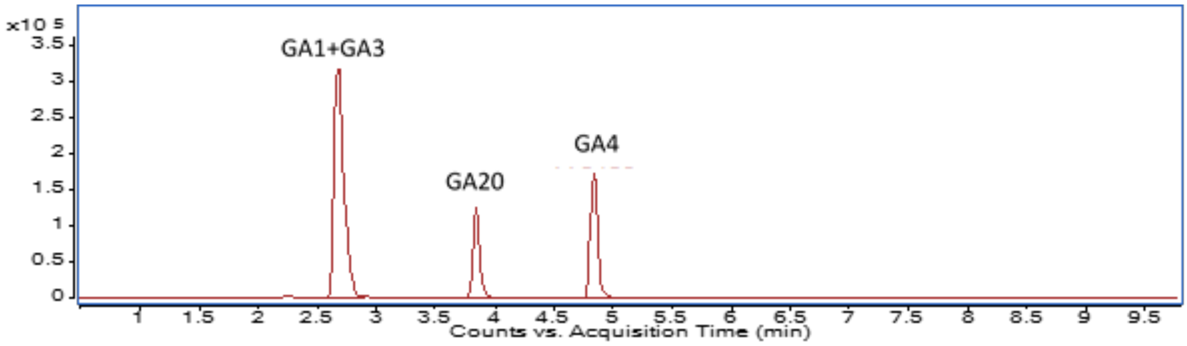

(b) Total ion chromatogram for trans-Zeatin and trans-Zeatin-riboside standards

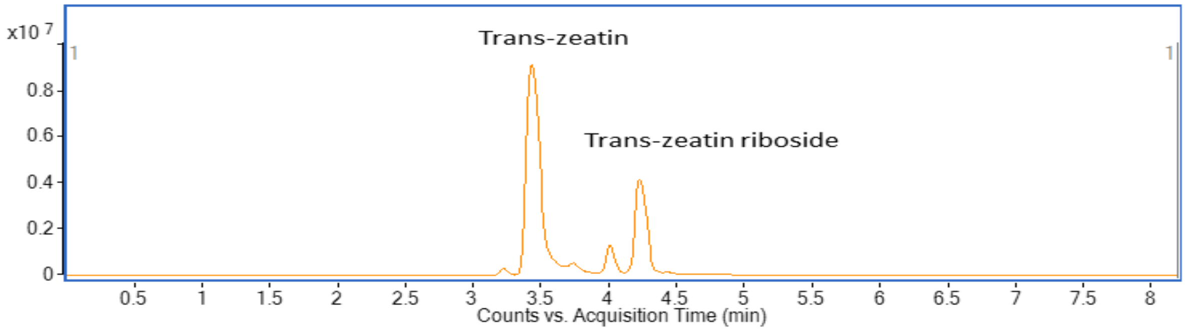

Supplementary Figure S4a-d:

Multiple Reaction Monitoring (MRM) for Gibberellins and Cytokinins, together with the precursor ions, and fragment ion transitions, respectively.

(a) Multiple Reaction Monitoring (MRM) transitions for GA_3 showing precursor ion and fragment ion transitions

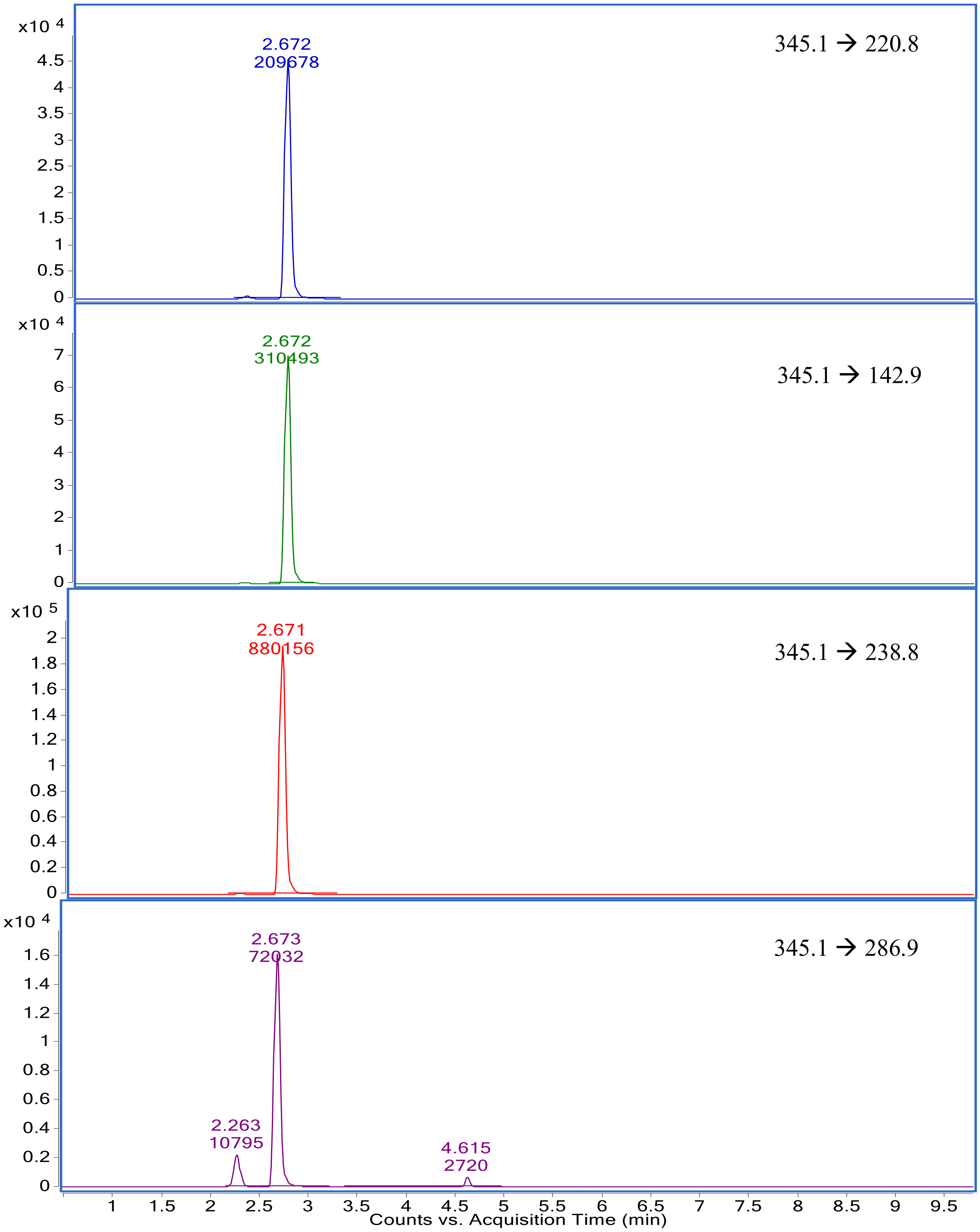

(b) MRM transitions for GA_1 showing precursor ion and fragment ion transitions

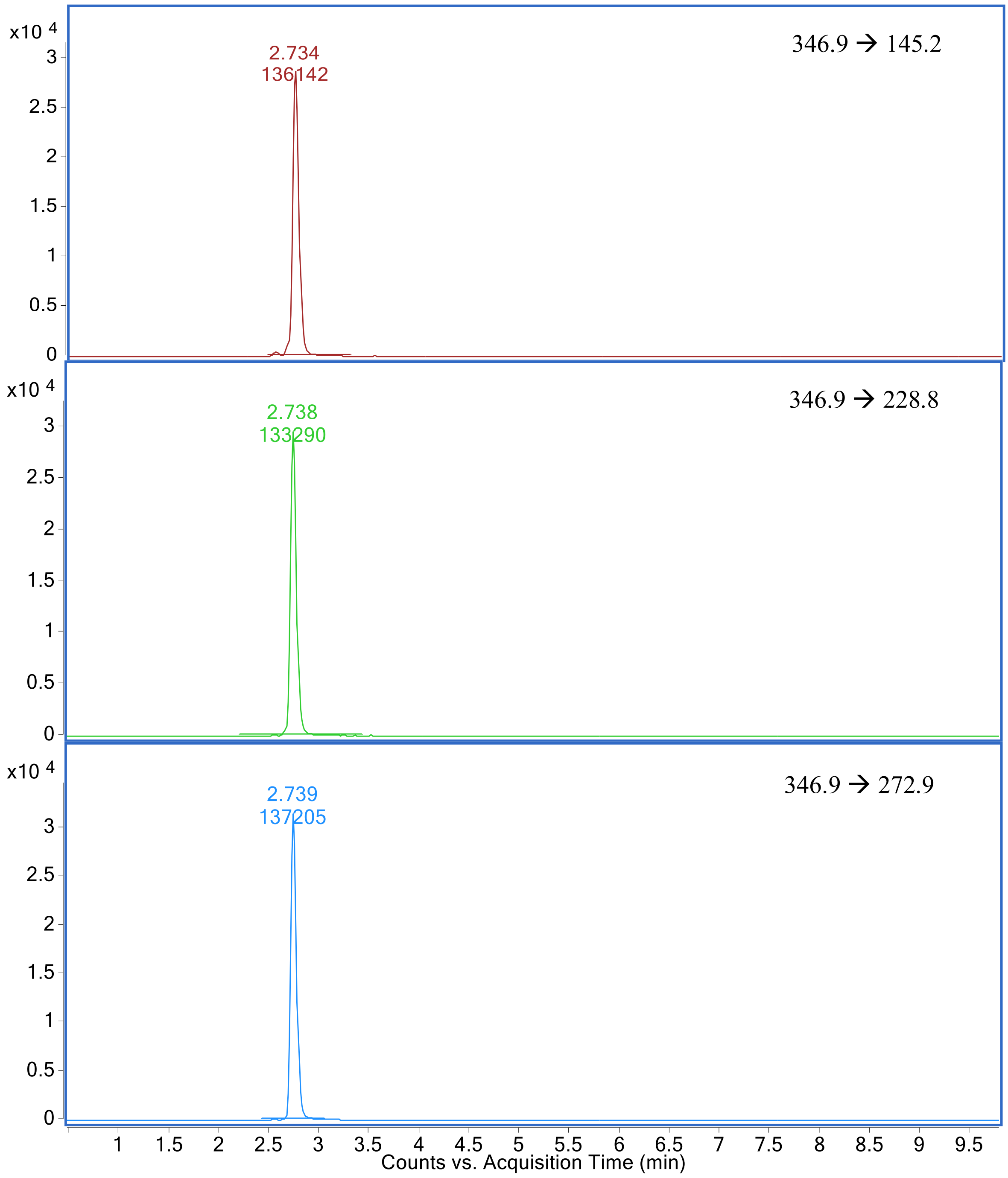

(c) MRM transitions for GA_4 showing precursor ion and fragment ion transitions

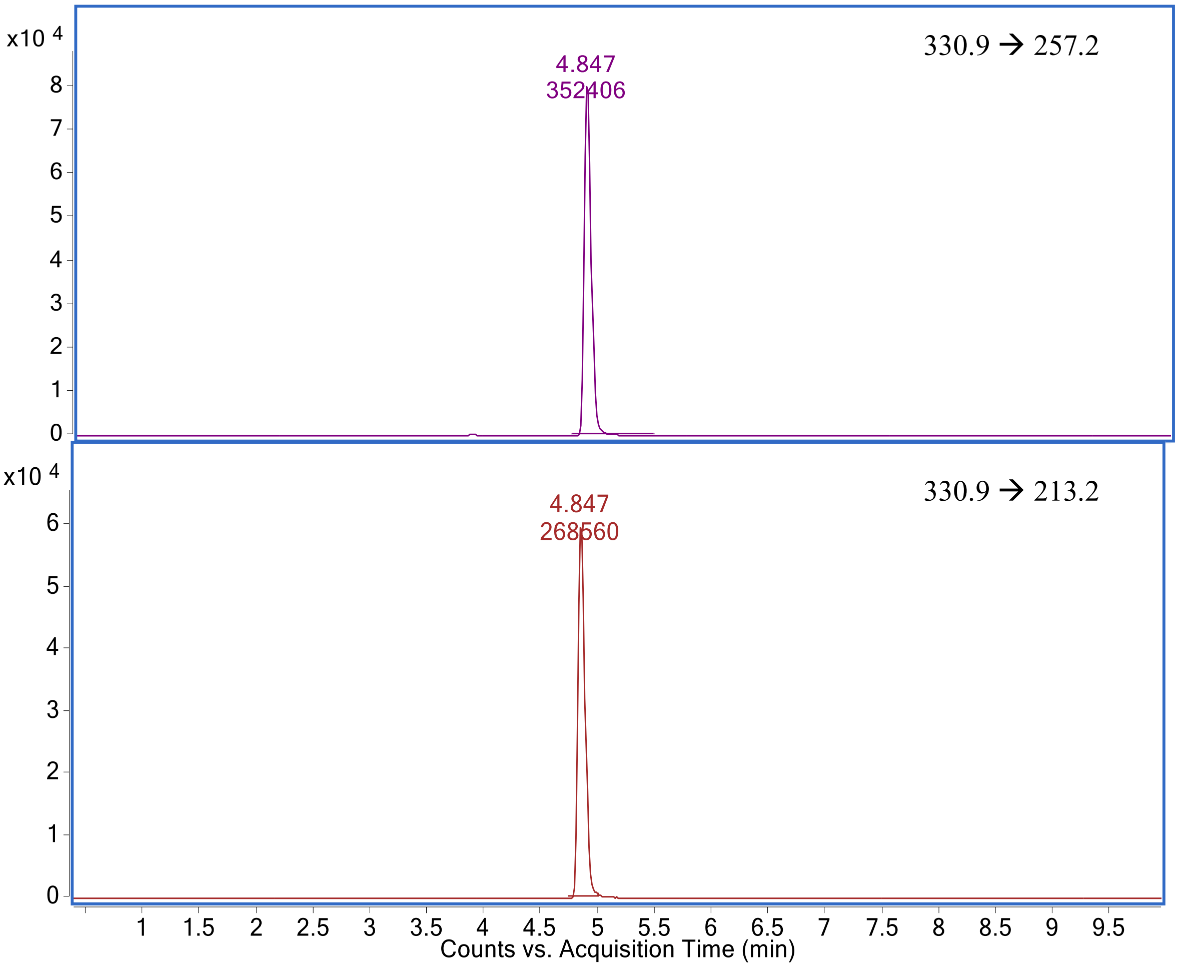

(d) MRM transitions for GA_{20} showing precursor ion and fragment ion transitions

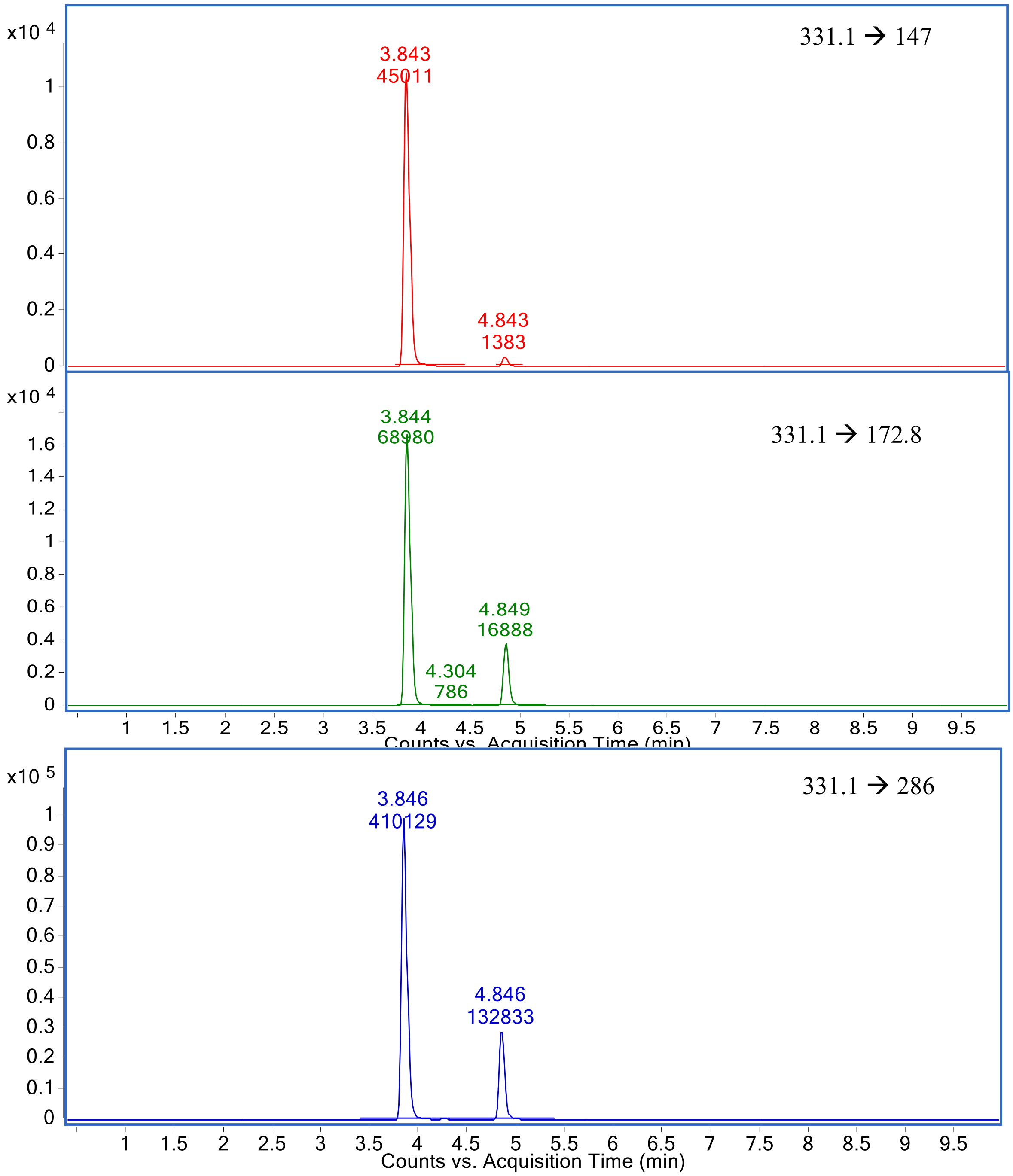

Supplementary Figure S5a-c:

MRM transitions for Cytokinins showing the respective precursor ion and the fragment ion transitions.

(a) MRM transitions for trans-Zeatin-riboside showing precursor ion and fragment ion transitions

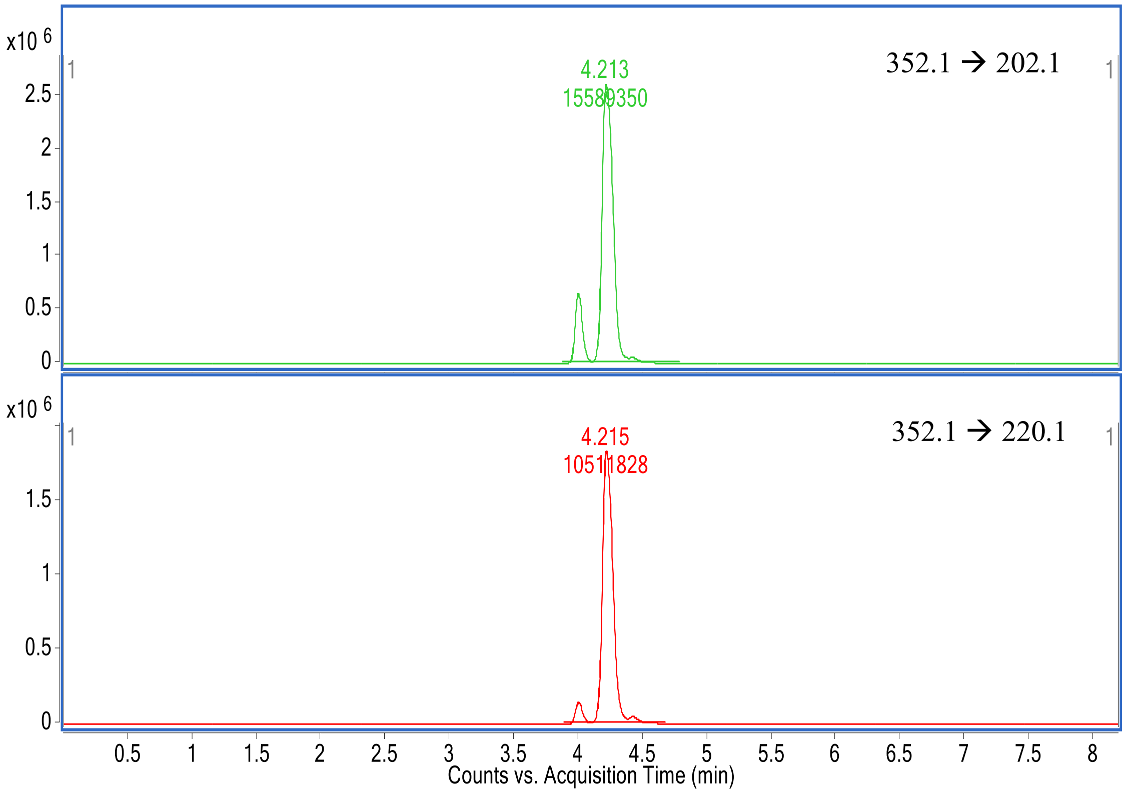

(b) MRM transitions for trans-Zeatin showing precursor ion and fragment ion transitions

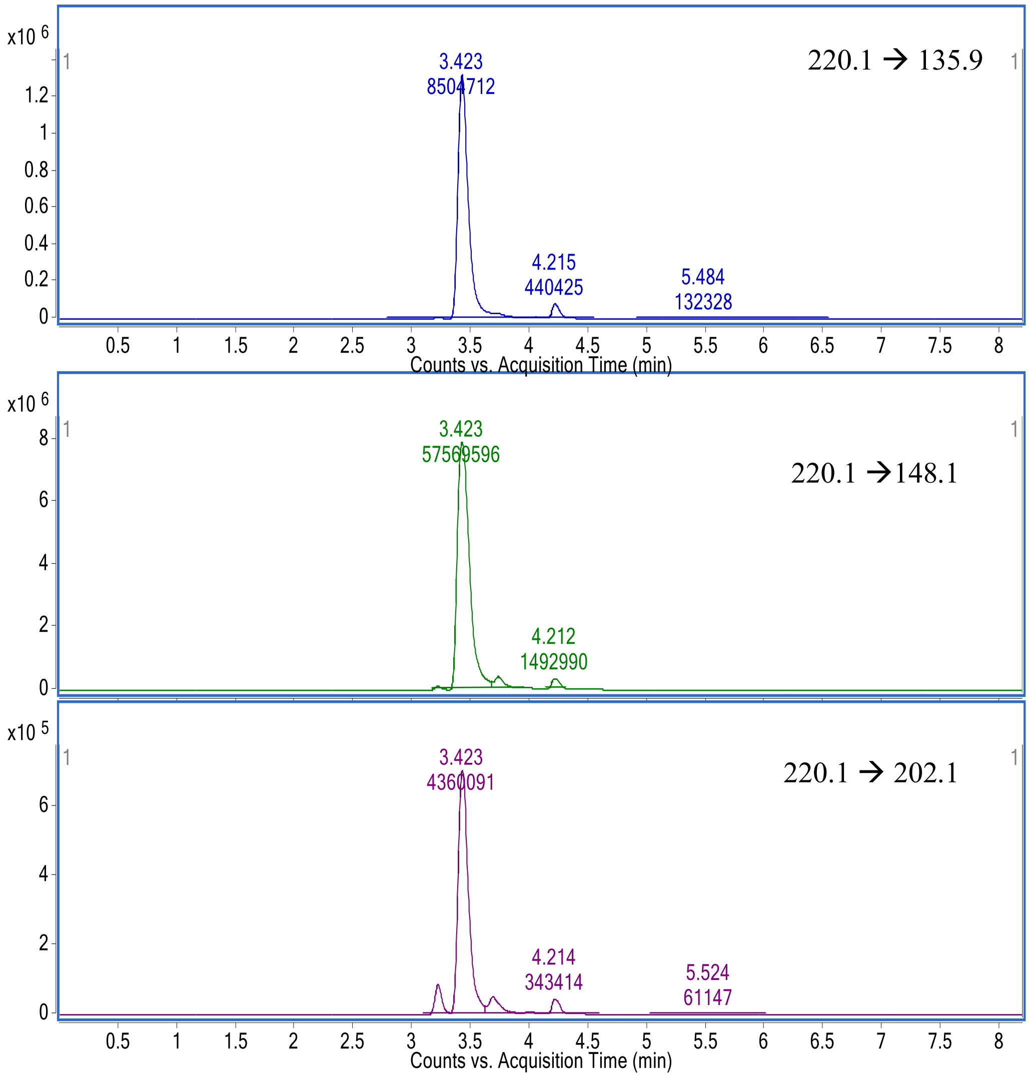

(c) Overlay Total Ion Chromatogram (TIC) between trans-Zeatin and trans-Zeatin-riboside standards (500 ppb each) and cell-free exudates of *P. citrinum* isolate B9 or FLP7 along with culture medium extract (Control; uninoculated). Trans-Zeatin and trans-Zeatin-riboside are present in culture filtrates of *P. citrinum* isolates (upper and lower panels) as well as in minor amounts in growth media (in blue).

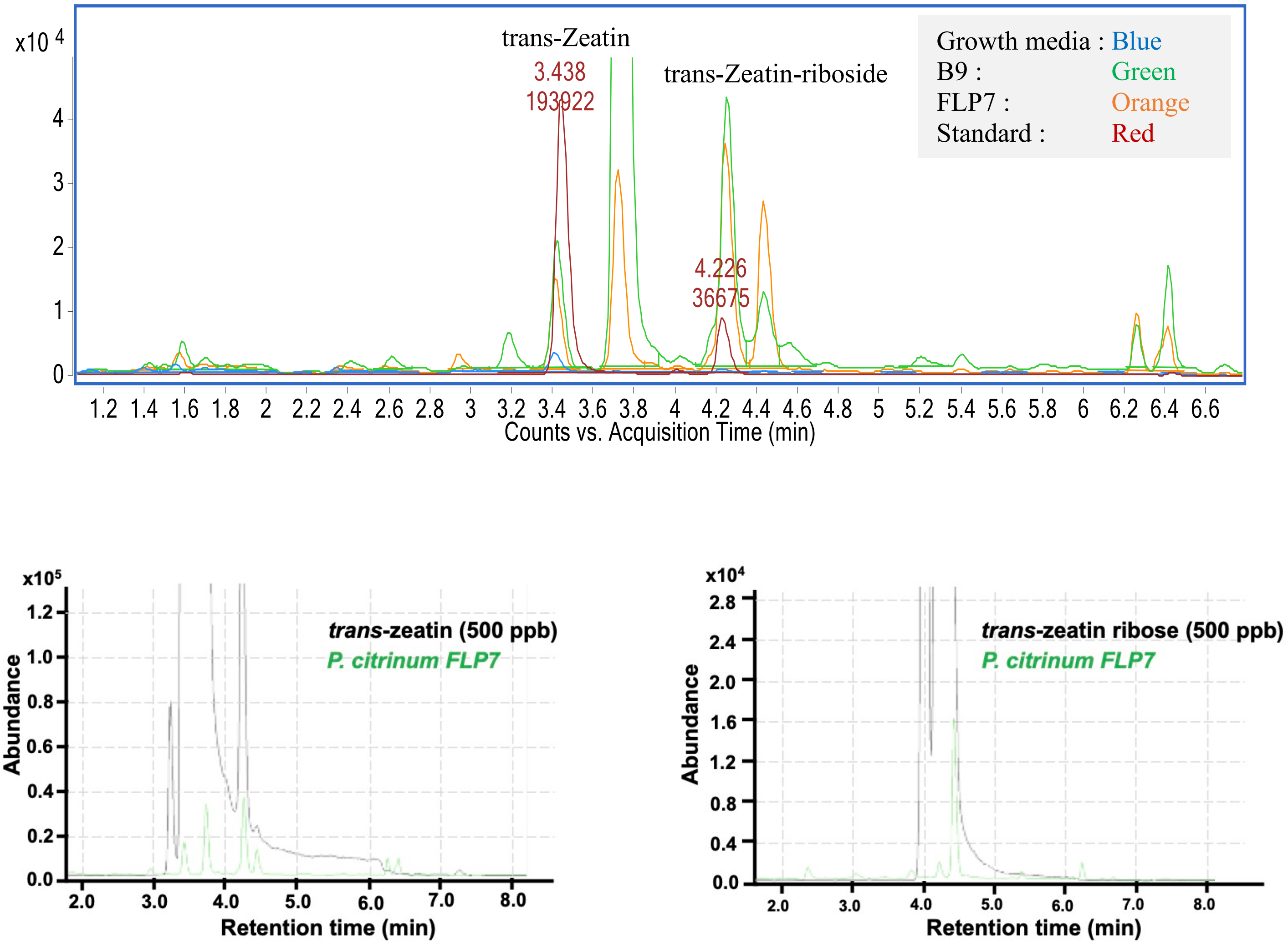

Supplementary Figure S6a-b

Overlay total ion chromatogram (TIC) for cell-free exudate of *P. citrinum* FLP7 isolate showing three specific Gibberellin variants.

(a) Overlay total ion chromatogram (TIC) for two replicates of *P. citrinum* FLP7 cell-free culture filtrates showing the presence of $GA_1 + GA_3$, and GA_{20}.

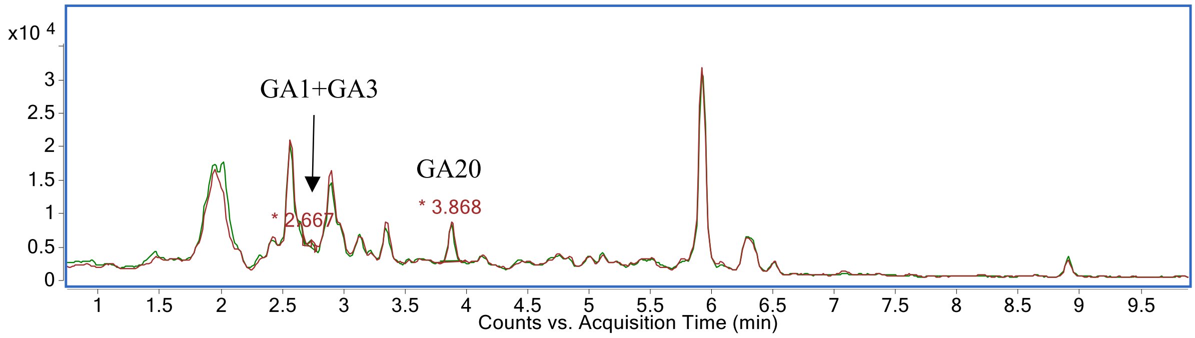

(b) Overlay total ion chromatogram (TIC) between GA_{20} standard and the cell-free culture filtrates of FLP7 isolate. It shows presence of GA_{20} in the culture filtrate.

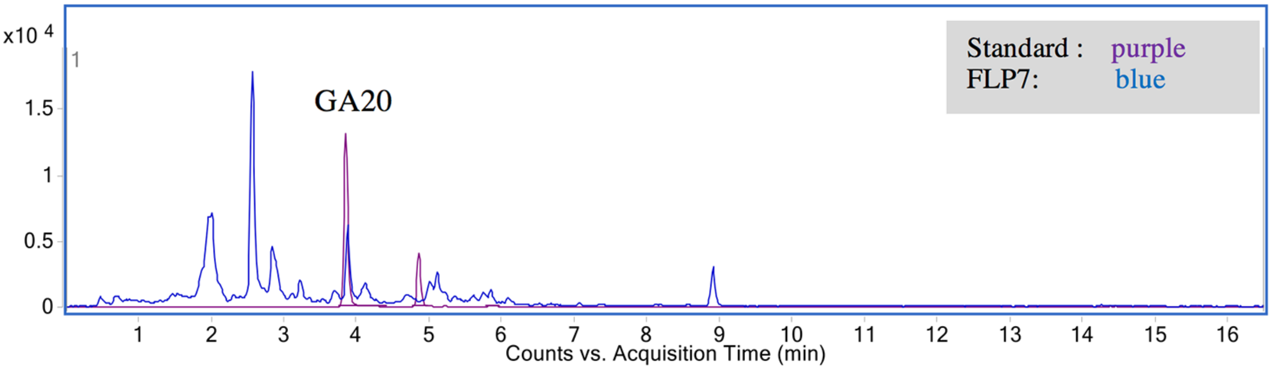

TABLE S1:

Oligonucleotide primers used in this study.

Name	Sequence (5'-3')	References
ITS1	TCCGTAGGTGAACCTGCGG	(White et al., 1990)
ITS4	TCCTCCGCTTATTGATATGC	(White et al., 1990)
eGFP-F1	TGGTGAGCAAGGGCGAGGAG	This study
eGFP-R1	CGTCCATGCCGAGAGTGATCC	This study
Hyg-F1	TCTCCGACCTGATGCAGCTCTC	This study
Hyg-R1	TACACAGCCATCGGTCCAGACG	This study
LSU-F	ACCCGCTGAACTTAAGC	(Schoch et al., 2012)
LSU-R	TCCTGAGGGAAACTTCG	(Schoch et al., 2012),
SSU-F	GTAGTCATATGCTTGTCTC	(Schoch et al., 2012)
SSU-R	CTTCCGTCAATTCCTTTAAG	(Schoch et al., 2012)

Table S2:

Selected reaction monitoring conditions for protonated or deprotonated forms of the indicated plant hormones ($[M+H]^+$ or $[M-H]^-$)

Compound Name	Retention Time (RT)	Precursor Ion (Q1)	Product Ion (Q3)	Collision Energy	Polarity
**Gibberellin GA_1**	2.73	346.9	272.9	32	Negative
		346.9	228.8	30	
		346.9	145.2	30	
**GA_3**	2.67	345.1	300.9	22	Negative
		345.1	238.8	22	
		345.1	220.8	22	
		345.1	142.9	22	
**GA_{20}**	3.84	331.1	286.9	30	Negative
		331.1	172.8	36	
		331.1	147	30	
**GA_4**	4.84	330.9	257.2	30	Negative
		330.9	213.2	32	
**Cytokinin / Trans-zeatin**	3.42	220.1	135.9	16	Positive
		220.1	202.1	15	
		220.1	148.1	15	
**Trans-zeatin riboside**	4.21	352.1	220.1	19	Positive
		352.1	202.1	19	

TABLE S3:

List of volatile organic compounds differentially emitted during *P. citrinum*-derived growth promotion of Choy Sum. The VOCs were detected using headspace-solid phase microextraction (HS-SPME) coupled with Gas chromatography mass spectrometry. Uninoculated Prune agar medium served as a negative/mock control for comparison.

Retention Time	Compound name	CAS number
7.036	Oxime-, methoxy-phenyl-_	NIST#: 222866
9.425	1-Octen-3-ol	CAS#: 3391-86-4
15.793	Methyl salicylate	CAS#: 119-36-8
27.569	1H-2-Indenone,2,4,5,6,7,7a-hexahydro-3-(1-methylethyl)-7a-methyl	CAS#: 5413-60-5
19.505	Decane, 2,3,5,8-tetramethyl-	CAS#: 124-18-5
21.604	Longifolene-(V4)	CAS#: 475-20-7
16.89	2-Allyl-4-methylphenol	CAS#: 6628-06-4
